# A new species of *Hedgpethia* (Arthropoda, Pycnogonida, Colossendeidae) from southwestern Japan

**DOI:** 10.3897/zookeys.175.2612

**Published:** 2012-03-16

**Authors:** Yoshie Takahashi, Hiroshi Kajihara, Shunsuke F. Mawatari

**Affiliations:** 1 Sapporo Daiichi High School, Sapporo 062-0021, Japan; 2 Faculty of Science, Hokkaido University, Sapporo 060-0810, Japan; 3 The Hokkaido University Museum, Sapporo 060-0810, Japan

**Keywords:** Pantopoda, Nansei Islands, TRV *Toyoshio-maru*, new species, taxonomy

## Abstract

We describe *Hedgpethia spinosa* **sp. n.** based on a single male specimen obtained from 197–207 m depth, south of Yaku Island, Kagoshima Prefecture, Japan. Among 15 previously known congeners, the new species resembles *Hedgpethia bicornis* (Losina-Losinsky & Turpaeva, 1958), *Hedgpethia chitinosa* (Hilton, 1943), and probably *Hedgpethia brevitarsis* (Losina-Losinsky & Turpaeva, 1958), in having a mid-dorsal tubercle on the posterior rim on each trunk segment. The new species, however, is distinguishable from those by a pair of horns on the anterior margin of the cephalic segment, spines on the first coxae, and denticulate spines on the strigilis. The new species represents the fifth member of the genus so far known from Japanese waters, in addition to *Hedgpethia brevitarsis* (Losina-Losinsky & Turpaeva, 1958), *Hedgpethia chitinosa* (Hilton, 1943), *Hedgpethia dofleini* (Loman, 1911), and *Hedgpethia elongata* Takahashi, Dick & Mawatari, 2007.

## Introduction

Pycnogonids have been taxonomically relatively well studied in Japan, beginning with Böhm’s (1879) report of two new species from Enoshima, Sagami Bay; about 160 species have now been recorded from Japanese waters (Miyazaki and Stock 1995, Nakamura 1995, Child 1996, Nakamura et al. 1996, Takahashi et al. 2007). While most studies have focused on the Pacific coast of central Japan, there have been fewer reports of pycnogonids from waters adjacent to the Nansei Islands, from where 23 species in 17 genera belonging to nine families have so far been recorded (Ohshima 1935, Nakamura and Child 1988, Child 1996, Takahashi et al. 2007).

The colossendeid genus *Hedgpethia* Turpaeva, 1973 contains 15 species (Arango 2009, Bamber 2010). All of these are fairly small in body size compared to most species of *Colossendeis*, the type genus of Colossendeidae. Species of *Hedgpethia* have been collected from a wide range of depths, from 20 m [*Hedgpethia chitinosa* (Hilton, 1943) (Utinomi 1971: 338)] to 4294 m [*Hedgpethia articulata* (Loman, 1908) (Turpaeva 1994: 135)]; most species have been collected infrequently, at only a few sites. From Japanese waters, four species of *Hedgpethia* have been reported so far, viz. *Hedgpethia brevitarsis* (Losina-Losinsky & Turpaeva, 1958) (Nakamura and Child 1983, 1991), *Hedgpethia chitinosa* (Hilton, 1943) (Hedgpeth 1949, Utinomi 1955, 1962, 1971, Nakamura and Child 1983, 1991, Nakamura 1987), *Hedgpethia dofleini* (Loman, 1911) (Loman 1911, Hedgpeth 1949, Utinomi 1951, 1955, 1971, Nakamura and Child 1991), and *Hedgpethia elongata* Takahashi, Dick and Mawatari, 2007 (Takahashi et al. 2007).

During a research cruise of the Training and Research Vessel *Toyoshio-maru*, Hiroshima University, in May 2005, a specimen of *Hedgpethia* was procured. We describe it as a new species in this paper.

## Material and methods

Collection, preparation, and measurements of the specimens primarily follow the methods of Takahashi et al. (2007). The voucher specimen has been deposited in the Hokkaido University Museum, Sapporo, Japan (ZIHU).

## Results

### Hedgpethia spinosa sp. n.

urn:lsid:zoobank.org:act:C228D530-5701-46F7-894F-EC12941C99C7

http://species-id.net/wiki/Hedgpethia_spinosa

Fig. 1

#### Material examined.

 Holotype: male, ZIHU 3335, 30°08.90'N, 130°38.04'E, south of Yaku Island, Kagoshima, 26 May 2005, 197–207 m depth, collected by plankton net in a beam trawl [inner net *sensu* Akiyama et al. (2008)], S. Ohtsuka leg.

#### Measurements of holotype (millimeters).

 Trunk length, 1.28; body width, 0.62; length of proboscis, 1.43; length of abdomen, 0.08; length of palp, 2.26; first article of palp (P1), 0.06; P2, 0.03; P3, 0.83; P4, 0.10; P5, 0.50; P6, 0.14; P7, 0.12; P8, 0.15; P9, 0.16; P10, 0.17; third leg, coxa 1, 0.21; coxa 2, 0.18; coxa 3, 0.16; femur, 1.33; tibia 1, 1.73; tibia 2, 1.52; tarsus, 0.56; propodus, 0.65; claw, 0.33; oviger, first article (O1), 0.04; O2, 0.11; O3, 0.11; O4, 1.20; O5, 0.21; O6, 1.18; O7,0.21; O8, 0.19; O9, 0.19; O10, 0.14.

#### Description.

**Figure 1.**
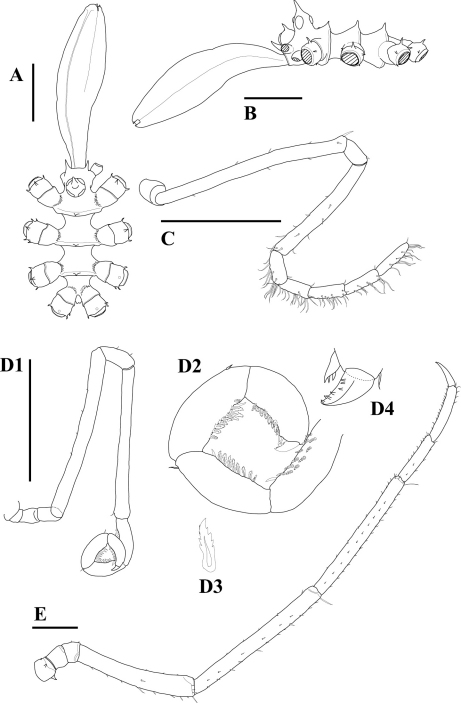
*Hedgpethia spinosa* sp. n. Holotype, male (ZIHU 3335). **A** trunk, dorsal view **B** trunk, lateral view **C** palp **D1** oviger **D2** enlargement of distal segments of oviger **D3** enlargement of denticulate spine constituting strigilis **D4** enlargement of terminal claw of oviger **E** left third leg. Scale bars: 0.5 mm.

 Size small for genus, leg span only 6.5 mm. Trunk (Fig. 1A, 1B) moderately short for genus, completely segmented, posterior rims of segments 1–3 inflated, each with pointed dorsal median tubercle. Lateral processes almost as long as their basal width, separated from one another by slightly more than their basal width, glabrous. Cephalic segment with pair of horn-like spines at anterior margin. Ocular tubercle dome shaped, 1.5 times as high as its basal width, with pointed apex projecting slightly forward. Eyes slightly pigmented, anterior pair larger than posterior pair. Proboscis (Fig. 1A, 1B) 1.2 times as long as trunk, swollen, spindle shaped, constricted at middle of total length, slightly curved downward, tapering distally; lips rounded, each with short ciliary sheet. Abdomen very small, located on ventral side.

Palps (Fig. 1C) longer than proboscis, slender; 10-segmented, with two short basal segments; first segment about twice as wide as other segments; second segment shortest; third segment longest, straight, with sparse, short setae, and with a few longer setae dorsodistally; fourth segment same length as sixth; fifth segment 0.6 times as long as third, with sparse setae over entire surface of distal half; seventh, eighth, and ninth segments subequal to sixth segment in length and slightly shorter than terminal segment; distal five segments fairly setose ventrally, setae as long as segment width, each segment with single short dorsodistal seta.

Oviger (Fig. 1D1) slender, long, 10-segmented; fourth and sixth segments longest, with very tiny setae ectally; fifth segment almost as long as second and third combined; strigilis (Fig. 1D2) armed with single short seta ectodistally, with rows of slender endal spines having denticles (Fig. 1D3); seventh segment equal to fifth in length; terminal segment less than two-thirds length and width of seventh segment; terminal claw short, about one-fifth as long as terminal segment, having small spines endally (Fig. 1D4).

Legs (Fig. 1E) slender, with many tiny setae over entire surface; first coxa with one small spine dorsally, one or two spines anteriorly and posteriorly, respectively; first and third coxae subequal and shorter than second coxa; femur almost equal to second tibia in length, curved ventrally, thickened in distal half, with several longer setae on distal end; tibia straight, with single long seta on distal end; first tibia 1.3 times as long as femur; tarsus slightly longer than propodus, both segments with dense, short setae ventrally and sparse, short setae dorsally; main claw about two-thirds as long as propodus.

#### Etymology.

 The specific name, a Latin adjective, refers to the spines on first coxae, anterior trunk margin, and terminal claw of oviger.

#### Remarks.

 Three species of *Hedgpethia* have pointed dorsomedian tubercles: *Hedgpethia bicornis* (Losina-Losinsky & Turpaeva, 1958), *Hedgpethia chitinosa* (Hilton, 1943), and probably *Hedgpethia brevitarsis* (Losina-Losinsky & Turpaeva, 1958), the tubercles of which are slightly rounded. However, none of these has a pair of horns on the anterior margin of the cephalic segment, spines on the first coxae, or denticulate spines on the strigilis. The anterior spines of the cephalic segment have the appearance of vestiges of chelifores. This is one of the smallest species in the genus.

## Supplementary Material

XML Treatment for Hedgpethia spinosa
